# Astrocytes regulate vascular endothelial responses to simulated deep space radiation in a human organ-on-a-chip model

**DOI:** 10.3389/fimmu.2022.864923

**Published:** 2022-08-30

**Authors:** Sonali D. Verma, Estrella Passerat de la Chapelle, Sherina Malkani, Cassandra M. Juran, Valery Boyko, Sylvain V. Costes, Egle Cekanaviciute

**Affiliations:** ^1^ Space Biosciences Division, National Aeronautics and Space Administration (NASA) Ames Research Center, Moffett Field, CA, United States; ^2^ Blue Marble Space Institute of Science, Seattle, WA, United States; ^3^ Bionetics, Yorktown, VA, United States

**Keywords:** central nervous system, spaceflight, ionizing radiation, astrocytes, organ models, neurovasculature

## Abstract

Central nervous system (CNS) damage by galactic cosmic ray radiation is a major health risk for human deep space exploration. Simulated galactic cosmic rays or their components, especially high Z-high energy particles such as ^56^Fe ions, cause neurodegeneration and neuroinflammation in rodent models. CNS damage can be partially mediated by the blood-brain barrier, which regulates systemic interactions between CNS and the rest of the body. Astrocytes are major cellular regulators of blood-brain barrier permeability that also modulate neuroinflammation and neuronal health. However, astrocyte roles in regulating CNS and blood-brain barrier responses to space radiation remain little understood, especially in human tissue analogs. In this work, we used a novel high-throughput human organ-on-a-chip system to evaluate blood-brain barrier impairments and astrocyte functions 1-7 days after exposure to 600 MeV/n ^56^Fe particles and simplified simulated galactic cosmic rays. We show that simulated deep space radiation causes vascular permeability, oxidative stress, inflammation and delayed astrocyte activation in a pattern resembling CNS responses to brain injury. Furthermore, our results indicate that astrocytes have a dual role in regulating radiation responses: they exacerbate blood-brain barrier permeability acutely after irradiation, followed by switching to a more protective phenotype by reducing oxidative stress and pro-inflammatory cytokine and chemokine secretion during the subacute stage.

## Introduction

Deep space radiation presents a critical health risk to astronauts on lunar and Mars missions. Beyond the magnetic field of the Earth, astronauts are predicted to be exposed to a dose of ionizing radiation primarily carried by the galactic cosmic rays (GCRs) of around 0.45 mGy per day, or 0.5 Gy per 3-year Mars mission. GCRs are composed of approximately 87% protons, 12% helium particles and 1% high Z-high energy (HZE) particles ranging from ^12^C to ^56^Fe, which have strong biological effects (1, 2). GCRs cause numerous health risks that currently lack mitigation strategies, including central nervous system (CNS) damage.

In animal models, exposure to simulated GCRs and HZE particles lead to cognitive and behavioral deficits correlated with neuroinflammation and neuronal damage (3–7). However, there is a major gap in research addressing the risk of *human* neurodegeneration and neuroinflammation caused by space radiation, especially in 3D organ models.

In addition to direct effects on the CNS, spaceflight and simulated GCRs particles cause chronic systemic immune dysfunction (8–10), which may exacerbate neuroinflammation and CNS damage *via* the blood-brain barrier (BBB). This interaction between CNS and the immune system is supported by the finding that peripheral immune cells can serve as a biomarker for behavioral deficits after HZE particle irradiation (11). Nonetheless, the effects of deep space radiation on the blood-brain barrier largely remain to be investigated in both human and in animal models.

Human organs-on-a-chip and organoids are multi-cellular three-dimensional structures that are increasingly used to evaluate the physiological, molecular and cellular effects of environmental stressors and diseases (12, 13). They are common in terrestrial biomedical research and are beginning to be adapted as flight payloads (14) with results to be determined. In addition, human induced pluripotent stem cell (iPSC)-derived cells can be used to seed organoids and organs-on-a-chip for personalized medicine approaches and for analyzing the variability of biological outcomes (15, 16). Organ-on-a-chip models can be cultured and tested at a much higher throughput (17), which is beneficial for evaluating a wide range of biological outcomes in response to different spaceflight stressors, making them highly suitable for our study focused on characterizing the impact of a range of ionizing radiation qualities, doses and timepoints.

*OrganoPlate™* is a commercially available (Mimetas, Inc.) organ-on-a-chip system that can be seeded with human iPSC-derived cells to form BBB models with leak-tight blood vessels. It allows high-throughput quantification of 45 – 96 samples per plate (18–21). OrganoPlates have been successfully used to quantify cellular and tissue-level responses to neurotoxic compounds and disease models (18, 19, 22).

We used OrganoPlates seeded with human iPSC-derived astrocytes and brain endothelial cells, or brain endothelial cells alone, as a model to investigate cellular and organ-level responses to 600 MeV/n ^56^Fe ions and simulated GCRs. We evaluated the permeability and morphology of vascular structures formed by endothelial cells, as well as oxidative stress and secreted cytokine and chemokine levels, in response to two radiation doses and sham control over 1-7 days after irradiation. Furthermore, we examined the role of astrocytes in mediating radiation responses by comparing models with and without astrocytes. Overall, our results describe a human CNS model suitable for automated payload adaptation in future space biology studies and suggest astrocyte regulatory mechanisms as targets for countermeasures to mitigate human BBB impairments during deep space exploration.

## Materials and methods

### Cell culture

Primary human endothelial cells were acquired from Millipore (hCMEC/D3, #SCC06) and Cell Biologics (PBMEC, # H-6023). hCMEC/D3 cells were cultured on plates coated with 5% collagen (Fisher #344702001) in PBS, in basal EndoGro media (Millipore #SCMEBM) with 0.5% FBS (VWR #1300-500H), 0.5% L-glutamine, 0.1% Pen/Strep (Millipore #516106), 0.02% LS-supplement, 0.01% EGF (ThermoFisher #PHG0311), 0.0001% FGF2 (ThermoFisher # PHG0359), 0.01% hydrocortisone hemisuccinate, 0.01% heparin sulfate, 0.01% ascorbic acid. All hCMEC/D3 media supplements without specified catalog numbers were acquired from Millipore. PBMEC cells were cultured on plates coated with gelatin (Fisher # 50-104-8358), in basal endothelial media (Fisher #50-104-8345) with 1% FBS (VWR #1300-500H), 0.2% endothelial cell supplement, 0.1% antibiotic solution, 0.1% L-glutamine, 0.01% hydrocortisone, 0.01% EGF (ThermoFisher #PHG0311), 0.01% FGF2 (ThermoFisher # PHG0359), 0.01% heparin, 0.01% VEGF. All PBMEC media supplements without specified catalog numbers were acquired from Cell Biologics.

Primary human iCell astrocytes were acquired from Cellular Dynamics (#01434) and cultured on plates coated with 5% Matrigel (Fisher #CB-40234A) in DMEM/Glutamax (ThermoFisher # 10569044) in DMEM/Glutamax (ThermoFisher # 10569044) with 1.5% FBS (VWR #1300-500H), 0.1% N2 supplement (ThermoFisher # 17502048), 0.1% non-essential amino acids, 0.1% Pen/Strep (Millipore #516106), 0.002% EGF (ThermoFisher #PHG0311), 0.002% FGF2 (ThermoFisher # PHG0359).

All cells were maintained in a humidified incubator (37°C, 5% CO_2_), and medium was replaced every 2-3 days. Cells were detached using 0.05% Trypsin (Gibco #15400), and all experiments were performed between passages 3 and 6 for all cell types.

### OrganoPlate seeding

Seeding of cells into 2-lane OrganoPlates (Mimetas, Inc.) was performed as previously described by Mimetas protocols. Briefly, astrocytes were harvested, resuspended at 7,000 cells/μL in 7mg/mL Matrigel solution (diluted in DMEM/Glutamax), and dispensed into gel inlets at a volume of 1.8μL. Plates were placed flat in a humidified incubator for 15 minutes to allow ECM gel polymerization, followed by an addition of 50μL of complete astrocyte medium to gel inlet wells. While endothelial cells were being prepared, plates were left on a rocker platform (7° tilt, 0.5 cycles/minute) in a humified incubator.

Endothelial cells were harvested, resuspended in their respective complete medium at 10,000 cells/μL, and dispensed into medium inlets at a volume of 2μL. An additional 50μL of complete endothelial cell medium was added to medium inlet wells. The plate was then tilted for 4 hours in a humidified incubator before adding 50μL of complete endothelial cell medium to medium outlet wells. Plates were moved to a rocker platform in a humidified incubator, and media was changed every 48 hours. hCMEC/D3 endothelial cells were cultured with iCell astrocytes for 600 MeV/n ^56^Fe irradiation, and PBMEC endothelial cells were cultured with iCell astrocytes for SimGCRSim irradiation. All OrganoPlate irradiations occurred 3 days after seeding.

### Irradiation

All irradiation experiments were conducted at the National Aeronautics and Space Administration (NASA) Space Radiation Laboratory, located in Brookhaven National Laboratory (Upton, NY). For 600 MeV/n ^56^Fe irradiation (170 keV/μm LET), two doses were used: 0.3 Gy and 0.82 Gy, as well as 0 Gy sham control. Irradiation took 2-5 minutes during which the cells were kept at room temperature in the beamline. For simplified simulated GCRs (SimGCRSim), two doses were used: 0.25 Gy and 0.5 Gy, as well as 0 Gy sham control. SimGCRSim consists of the following particles delivered in sequence: 35% 1000 MeV/n ^1^H, 1% 600 MeV/n ^28^Si, 18% 250 MeV/n ^4^He, 6% 350 MeV/n ^16^O, 1% 600 MeV/n ^56^Fe and 39% 250 MeV/n ^1^H. Irradiation took 0.5 – 1 hour, during which the samples were kept in a cell culture incubator in the beamline.

### Barrier permeability assay

To assess the permeability of the vascular endothelial cell barrier, media in inlet and outlet wells was aspirated followed by flash-freezing for further supernatant assays and replaced with 50 μl endothelial cell media with 2% FITC-conjugated 40 kDa dextran (Sigma #FD40S-100MG) and 2% TRITC-conjugated 155 kDa dextran (Sigma #T1287-50MG). Images were collected every 5 minutes for 30 minutes total on Zeiss AxioVision microscope with 30 ms exposure for FITC (LED Calibri lamp, GFP filter) and 300 ms exposure for TRITC (LED Calibri lamp, DsRed filter), using 10x magnification, a single image per well per timepoint. N=8 wells per timepoint per irradiation per condition (with or without astrocytes) were imaged.

**Figure 1:**
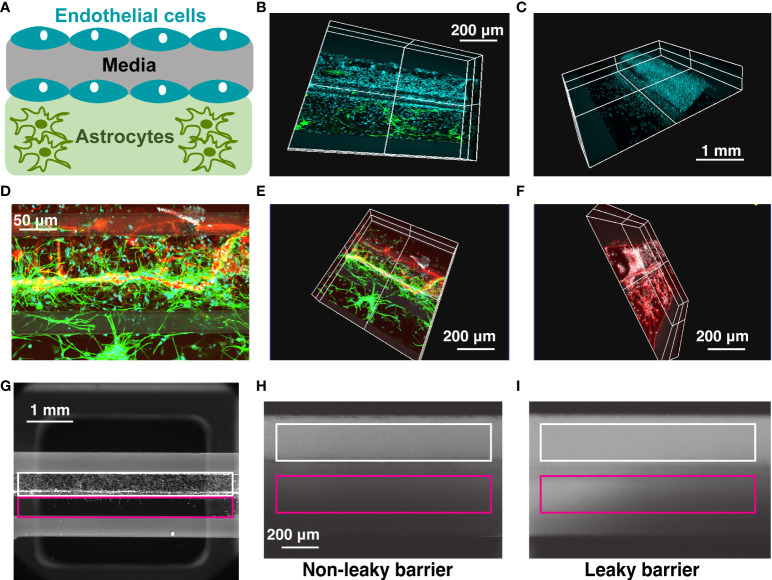
OrganoPlate blood-brain barrier model. **(A)** Schematic representation of cells seeded in 2-lane OrganoPlate. Top lane, endothelial cells. Bottom lane, astrocytes. **(B–F)**. Representative images of OrganoPlates seeded with astrocytes and endothelial cells 10 days after plating. **(B)** Cyan, DAPI^+^ endothelial cell nuclei in the top lane. Green, GFAP^+^ astrocytes in the bottom lane. **(C)** Cyan, DAPI^+^ endothelial cell nuclei. **(D, E).** Red, PECAM^+^ endothelial cells in the top lane. Green, GFAP^+^ astrocytes in the bottom lane. **(F)** White, DAPI^+^ endothelial cell nuclei in the top lane. Red, GFAP^+^ astrocytes in the bottom lane. **(G–I)**. Representative images of a 2-lane OrganoPlate chip seeded with endothelial cells in the top lane (left), a chip with a non-leaky barrier (middle) and a chip with a leaky barrier (right) loaded with a fluorophore-conjugated dextran. White, endothelial cell channel. Magenta, extracellular matrix channel. Leaky barrier increases the relative fluorescence of dye in the extracellular matrix channel, compared to endothelial cell channel.

Images were quantified by measuring the ratio between the fluorescent signal in the ECM channel and in the lumen channel over time using ImageJ (FiJi version 1.53c). Regions of interest (ROI) of the exact same area were created for both channels (**Figure 1**), and the distance between the ROI box and the channel divider was kept consistent between images. The relative fluorescence was calculated using the average intensity of fluorescence in both channels at each timepoint:


$$Relative\;fluorescence=\frac{Mean\;fluorescence\;in\;ECM\;channel}{Mean\;\;fluorescence\;in\;lumen\;channel}$$

Next, the area under the resulting plotted curve was calculated using the trapezoidal method, which uses the sum of the area of each trapezoid between two time points as an approximation of the definite integral. The resulting relative area under the curve is directly proportional to the barrier permeability.

### Immunohistochemistry

Immunohistochemistry on OrganoPlates was performed as described by Mimetas protocols. Briefly, OrganoPlate culture chips were fixed with ice cold 100% methanol for 10-15 min at room temperature (RT). Each chip was washed once with 4% Fetal Bovine Serum (FBS; VWR #89501-186) in Phosphate Buffer Saline (PBS) for 5 min at RT, followed by permeabilization with 0.3% Triton X-100 (Sigma #T8787) in PBS for 10 min at RT. Chips were washed again with 4% FBS for 5 min at RT, and blocked in a buffer of 2% FBS, 2% Bovine Serum Albumin (BSA) (Sigma #A9647), and 0.1% Tween-20 (Sigma #P1379) in PBS for 45 min at RT. The cultures were then incubated overnight on a rocker platform (7° incline, 0.5 cycles/minute) at RT with primary antibodies diluted in blocking buffer.

The following primary antibodies were used for immunofluorescence: anti-platelet endothelial cell adhesion molecule-1 (PECAM-1; mouse, 1:20, Dako #M0823), anti-tight junction protein-1 (ZO-1; rabbit, 1:400, Invitrogen #61-7300), anti-glial fibrillary acidic protein (GFAP; goat, 1:1500, Abcam #ab53554) and anti-aquaporin-4 (AQP4, rabbit, 1:200, Invitrogen #PA5-53234).

The following day chips were washed twice with 4% FBS for 3 minutes each, followed by incubation on a rocker platform with secondary antibodies in blocking buffer for 1 hour at RT. Cultures were again washed twice with 4% FBS for 3 minutes each. Nuclear counterstaining was performed using 4′,6-diamidino-2-phenylindole (DAPI; 1:2000 in PBS, ThermoFisher #62248) for 30 minutes at RT on a rocker platform. Samples were washed once with PBS for 5 minutes and stored at 4°C in PBS. The following secondary antibodies were used for detection of primary antibodies: AF488 donkey anti-goat (Invitrogen #A32814), AF594 donkey anti-rabbit (Invitrogen #A32754), AF647 donkey anti-mouse (Invitrogen #A32787), AF488 goat anti-rabbit (Invitrogen #A11034), and AF594 goat anti-mouse (Invitrogen #A11005).

Fluorescence images were acquired on an Olympus IX83 inverted confocal microscope using Olympus FluoView FV31S-SW Viewer Software (ver.2.6). Images were processed in ImageJ (version 2.1.0/1.53c) by quantifying the average fluorescence of the sum of the images in Z-stack in ROIs in endothelial cell channel (ZO1, PECAM1) or astrocyte channel (GFAP, AQP4.)

### 8-oxo-dG quantification

8-hydroxy-2’-deoxyguanosine (8OHdG) was quantified in OrganoPlate supernatants acquired from endothelial cell channels (media inlets and outlets) using 8-oxo-dG ELISA kit (R&D Systems, #4380-096K) according to manufacturer’s instructions. Supernatants were diluted in assay diluent 1:5 before loading. N = 12 samples were used per irradiation per timepoint per condition (with or without astrocytes).

### Immune cytokine and chemokine quantification

Immune cytokines and chemokines were quantified using the Milliplex Human Immune Cytokine/Chemokine Magnetic Bead Panel (Millipore, #HCYTMAG-60K) on MAGPIX multiplex Luminex system (Millipore, #80-073). Supernatants were diluted in assay diluent 1:2 before loading. N = 9-10 samples were used per irradiation per timepoint per condition (with or without astrocytes).

### Statistical analysis

In experiments with two independent variables (e.g. radiation and dose and astrocyte presence), 2-way ANOVA was used. For single variable analysis, 1-way ANOVA was used to compare >2 groups and Student’s t-test was used to compare 2 groups. P value of < 0.95 was considered significant. Analysis and graphical representation of results was done using GraphPad Prism (v. 9.1.0.)

## Results

### Human organ-on-a-chip model for investigating the effects of simulated deep space radiation on the BBB

We developed a high-throughput BBB organ-on-a-chip model using a Mimetas 2-lane OrganoPlate system that consists of 96 individual chips combined in a plate which follows a standard 384-well footprint (18). Each chip was composed of a media channel seeded with human brain endothelial cells, located on top of the extracellular matrix channel, which was either kept empty or seeded with human astrocytes (**Figure 1A**). Endothelial cells formed a 3D tubular structure by 3 days after seeding, which persisted for at least 10 days after seeding (**Figures 1B–F**), while astrocytes formed a tightly connected network and extended their processes towards the endothelial cell channel (**Figures 1B, D–F**). For quantification of barrier permeability, fluorophore-tagged 155 kDa and 40 kDa dextrans were loaded in the vascular channel (**Figures 1G–I**, white) and the relative fluorescence of dye leaking towards the neighboring extracellular matrix channel (**Figures 1G–I**, magenta) was measured as described in **Materials and Methods**.

**Figure 2:**
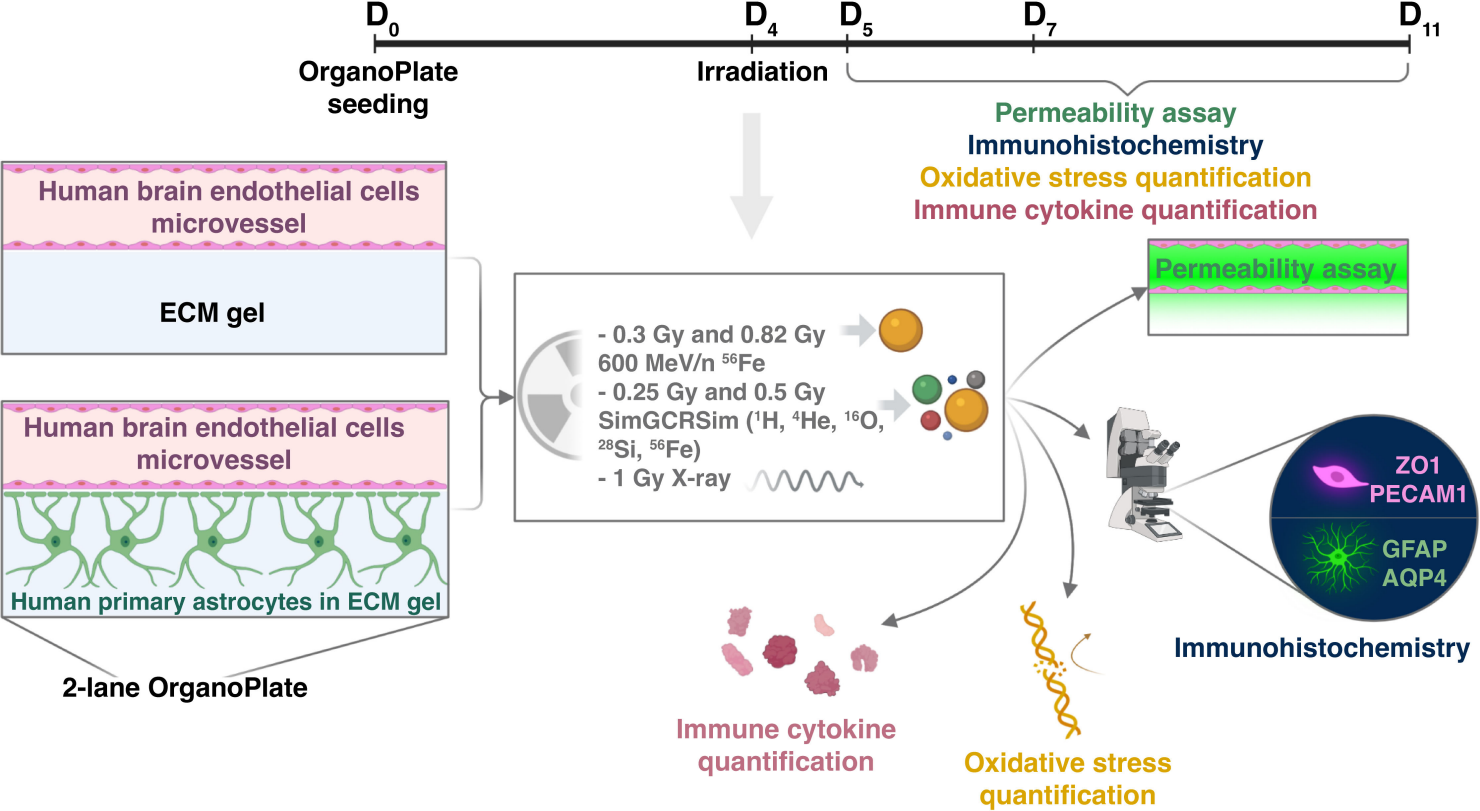
Experimental design. (Created with BioRender.com).

Three days after seeding, OrganoPlates were exposed to 5-ion simplified simulated galactic cosmic rays (SimGCRSim) (23) or their HZE components, 600 MeV/n ^56^Fe particles. The experimental design is depicted in **Figure 2**. These particles were selected due to their major biological effects on rodent CNS *in vivo* (24–26) combined with a gap in knowledge regarding their functions in human CNS and the associated blood-brain barrier effects in either animal or human models.

For consistency with previous work our lab and others have done on simulating deep space radiation, we chose a similar radiation dose regimen (27, 28). The dose response to ionizing radiation and the time course of resulting impairments were mapped by selecting two doses per radiation quality: 0.25 Gy and 0.5 Gy SimGCRSim, and 0.3 Gy and 0.82 Gy 600 MeV/n ^56^Fe, and analyzing outcomes at 1, 3 and 7 days post irradiation, which are the key time points for studying acute and subacute astrocytic and endothelial responses to terrestrial CNS injuries (29, 30). We quantified major physiological outcomes, including vascular endothelial cell barrier permeability, morphological changes, oxidative stress and secreted pro- and anti- inflammatory cytokines. In this study, we primarily focus on 600 MeV/n ^56^Fe responses, noting the similarities with SimGCRSim irradiation and other ionizing radiation qualities where relevant.

### Simulated deep space radiation causes BBB damage that is exacerbated by astrocytes 1 day post-exposure

Terrestrial CNS injuries, especially traumatic brain injury and stroke, increase blood-brain barrier permeability that is exacerbated by pro-inflammatory mediators secreted by astrocytes during the acute phase of injury response, beginning at 3 – 4 hours and lasting through 48 – 72 hours after injury (29, 31). To assess whether simulated deep space radiation has a similar effect, we exposed BBB OrganoPlates to SimGCRSim or 600 MeV/n ^56^Fe particles and quantified vascular endothelial permeability at 24 hours after irradiation. Relative fluorescence was expressed as area under the curve on a scale from 0 to 1, where 1 indicates complete permeability.

**Figure 3:**
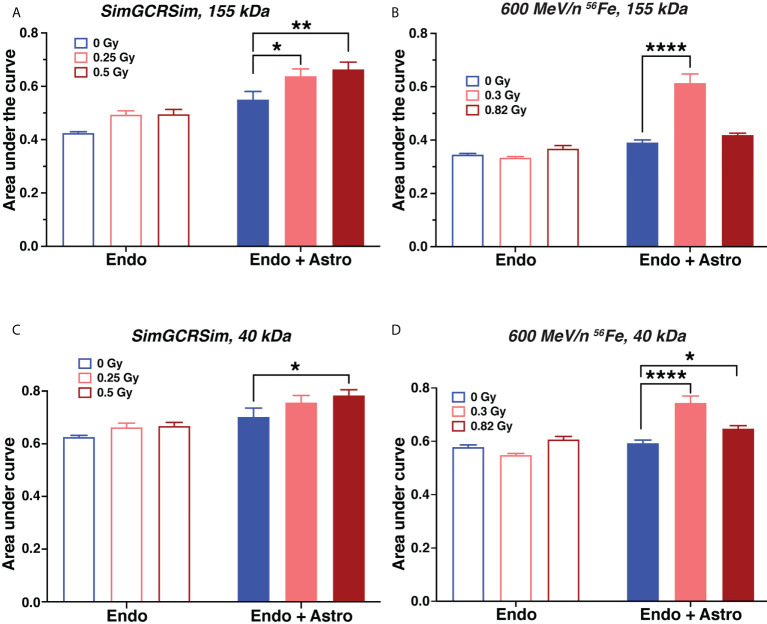
Astrocytes exacerbate acute endothelial cell barrier permeability caused by simulated deep space radiation. **(A–D)**. Vascular endothelial cell structure permeability 1 day after irradiation with 5-ion SimGCRSim **(A, C)** or 600 MeV/n ^56^Fe particles **(B, D)**, quantified as area under the curve of relative fluorescence of dye. Dye, TRITC-conjugated 155 kDa dextran **(A, B)** or FITC-conjugated 40 kDa dextran **(C, D)**. Endo, endothelial cells alone. Endo + astro, endothelial cells and astrocytes. Open bars, chips with endothelial cells only. Shaded bars, chips with endothelial cells and astrocytes. Blue, 0 Gy sham irradiation. Light red, lower dose (0.25 Gy SimGCRSim, 0.3 Gy 600 MeV/n ^56^Fe). Dark red, higher dose (0.5 Gy SimGCRSim, 0.82 Gy 600 MeV/n ^56^Fe). N = 8 chips per condition. Error bars, mean ± SEM. *p < 0.05, **p < 0.01, ****p < 0.0001, Dunnett’s multiple comparison test, 2-way ANOVA. Non statistically significant changes are not marked.

We observed that both SimGCRSim and 600 MeV/n ^56^Fe particles significantly increased vascular permeability to 155 kDa and 40 kDa fluorescent dextrans, which was exacerbated by astrocyte presence (**Figures 3A–D**). This result was recapitulated by findings using the same model in response to 1 Gy X-rays (**Supplementary Figures 1A–D**) and 600 MeV/n ^56^Fe particles in another series of experiments (**Supplementary Figures 1E, F** **)**, both of which increased the permeability of vascular structures formed by human brain endothelial cells to 155 kDa or 40 kDa fluorescent dextrans. No major differences have been observed in hCMEC/D3 and PBMEC endothelial cell responses. Notably, the observed dose response was not always linear, which is consistent with previously published non-linear effects of particle radiation on rodent CNS *in vivo* (32).

Barrier permeability to 40 kDa dextran (**Figures 3C, D**) was only slightly higher than to 155 kDa dextran (**Figures 3A, B**), indicating that ionizing radiation leads to comparatively large openings in the blood-brain barrier that are sufficient for 155 kDa protein to pass through. In addition, we observed that astrocytes increased the barrier permeability with or without radiation. We hypothesize that this effect is mostly likely caused by the physical disruption of the gel by astrocyte processes.

**Figure 4:**
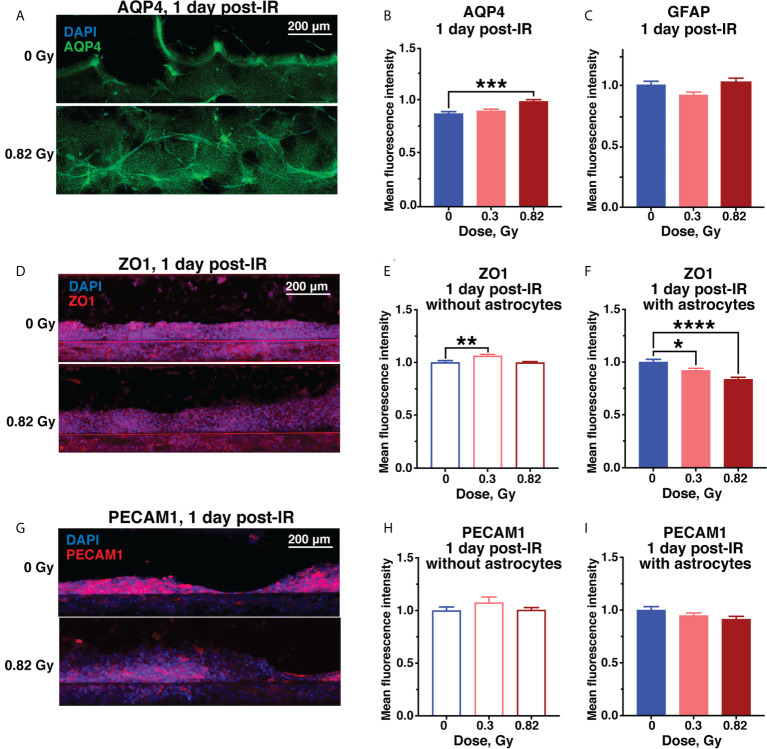
Acute astrocyte and endothelial cell damage from 600 MeV/n ^56^Fe irradiation. Representative images **(A)** and quantification **(B)** of AQP4 (green) immunofluorescence. **(C)** Quantification of GFAP immunofluorescence in the same chips as **(B)**. **(D–F)**. Representative images **(D)** and quantification of ZO1 (red) immunofluorescence in chips with endothelial cells only **(E)** and with endothelial cells and astrocytes **(F)**. **(G–I)**. Representative images **(G)** and quantification of PECAM1 (red) immunofluorescence in chips with endothelial cells only **(H)** and with endothelial cells and astrocytes **(I)**. All images counterstained with DAPI^+^ nuclei (blue). Open bars, chips with endothelial cells only. Shaded bars, chips with endothelial cells and astrocytes. Blue, 0 Gy sham irradiation. Light red, lower dose (0.3 Gy). Dark red, higher dose (0.82 Gy). N = 12-24 areas from 6-12 chips per condition. Error bars, mean ± SEM. *p < 0.05, **p < 0.01, ***p < 0.001, ****p < 0.0001, 1-way ANOVA, Dunnett’s multiple comparisons test. Non statistically significant changes are not marked.

Radiation-dependent increase in vascular permeability in models containing astrocytes was associated with increased levels of aquaporin-4 (AQP4) based on relative fluorescence (**Figures 4A, B**), while reactive astrocyte marker glial fibrillary acidic protein (GFAP) was not affected (**Figure 4C**). AQP4 is an astrocyte endfeet protein that regulates blood-brain barrier permeability and is similarly increased in rodent CNS in response to spaceflight (33) and aging (34).

The endothelial tight junction marker zonula occludens protein 1 (ZO1) was significantly reduced by simulated space radiation in a dose-dependent manner only in astrocyte-containing models, while it was slightly, but significantly increased in models without astrocytes (**Figures 4D–F**). The main role of ZO1 is limiting vascular barrier permeability, thus, this radiation and astrocyte-dependent ZO1 reduction is consistent with the observed increase in permeability to fluorescent dextrans. A similar decrease in ZO1 has been observed in response to spaceflight *in vivo* (33) and in neurodegenerative diseases associated with blood-brain barrier disruption (35). Notably, the persistent ZO1 reduction was specific to 600 MeV/n ^56^Fe particle irradiation and was not observed in response to SimGCRSim irradiation, where ZO1 was increased instead, indicating an alternate mechanism of radiation-mediated vascular permeability (**Supplementary Figures 2A, B** **)**.

Finally, the endothelial cell marker platelet and endothelial cell adhesion molecule 1 (PECAM1) was not significantly affected by radiation dose or astrocyte presence (**Figures 4G–I**), however, we observed a downwards trend in response to 0.82 Gy of 600 MeV/n ^56^Fe particle radiation (**Figure 4I**) that was significant in response to SimGCRSim (**Supplementary Figures 2C, D** **)**. In terrestrial CNS disorders, reduced PECAM1 has been associated with vascular permeability (36), while its increase is linked with neuroinflammation (37).

### Subacute responses to simulated deep space radiation include astrocyte activation and endothelial damage 3-7 days after exposure

**Figure 5:**
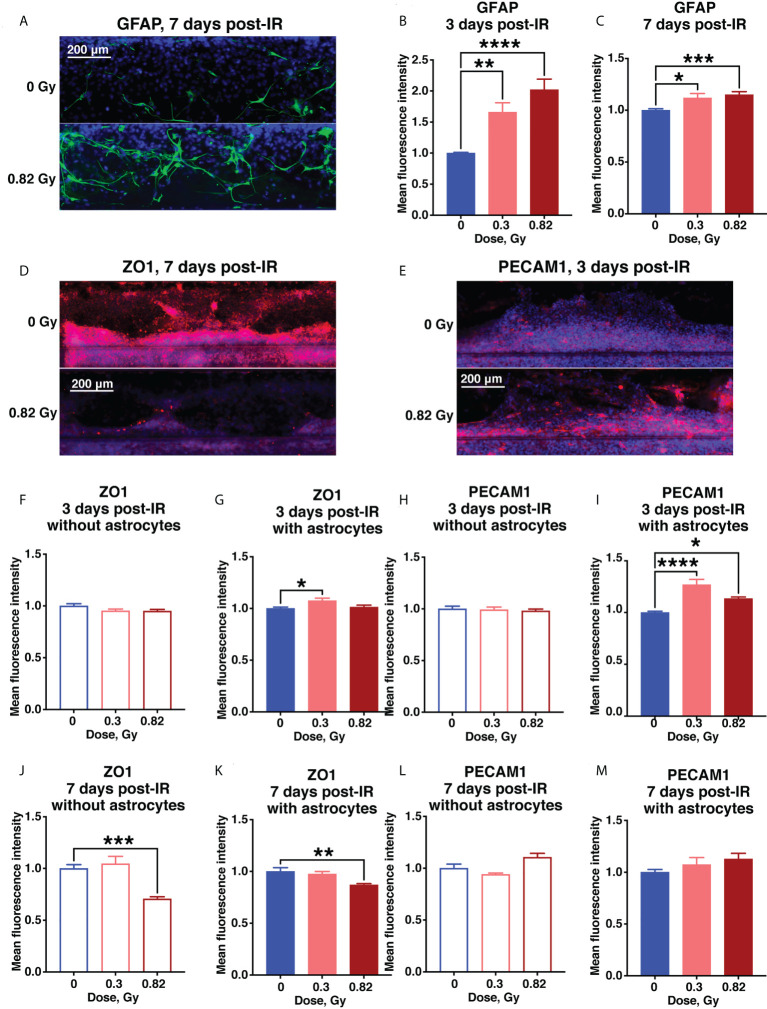
Subacute BBB damage from 600 MeV/n ^56^Fe irradiation is in part mediated by activated astrocytes. **(A–C)** Representative images **(A)** and quantification **(B, C)** of astrocytic GFAP (green) immunofluorescence 3 days **(B)** and 7 days **(C)** after irradiation. **(D–M)** .Representative images **(D, E)** and quantification **(F–M)** of endothelial ZO1 (red) and PECAM1 (red) immunofluorescence 3 days **(F–I)** and 7 days **(J–M)** after irradiation. All images counterstained with DAPI, blue. Open bars, chips with endothelial cells only. Shaded bars, chips with endothelial cells and astrocytes. Blue, 0 Gy sham irradiation. Light red, lower dose (0.3 Gy). Dark red, higher dose (0.82 Gy). N = 12-24 areas from 6-12 chips per condition. Error bars, mean ± SEM. *p < 0.05, **p < 0.01, ***p < 0.001, ****p < 0.0001, 1-way ANOVA, Dunnett’s multiple comparisons test. Non statistically significant changes are not marked.

In contrast to 24 hours after irradiation, by 3 days after irradiation the astrocytes began exhibiting the classic GFAP^+^ reactive phenotype, which persisted for at least 7 days after irradiation (**Figures 5A–C**), resembling subacute and chronic astrocytic scarring that occurs on approximately the same timescale after CNS injury or stroke (29). Ongoing tight junction and endothelial cell damage was indicated by a 600 MeV/n ^56^Fe-mediated reduction in ZO1 by 7 days after irradiation in samples with or without astrocytes, and an increase in PECAM1 that was significant at 3 days, but drifted back towards sham levels by 7 days after irradiation (**Figures 5D–M**, **Supplementary Figures 2E–H**). In combination, these result suggest that simulated deep space radiation induced a prolonged disruption of tight junctions, partially reduced by activated astrocytes.

### Simulated deep space radiation induces oxidative stress and inflammation that are partially mediated by astrocytes in the subacute stage after irradiation

To analyze the potential mechanisms underlying simulated deep space radiation-mediated BBB damage, we quantified secreted 8-oxo-deoxyguanosine (8-oxo-dG) as a marker of oxidative stress, as well as a panel of 38 pro- and anti-inflammatory cytokines and chemokines in the supernatant collected from the vascular channel at different time points after irradiation.

**Figure 6:**
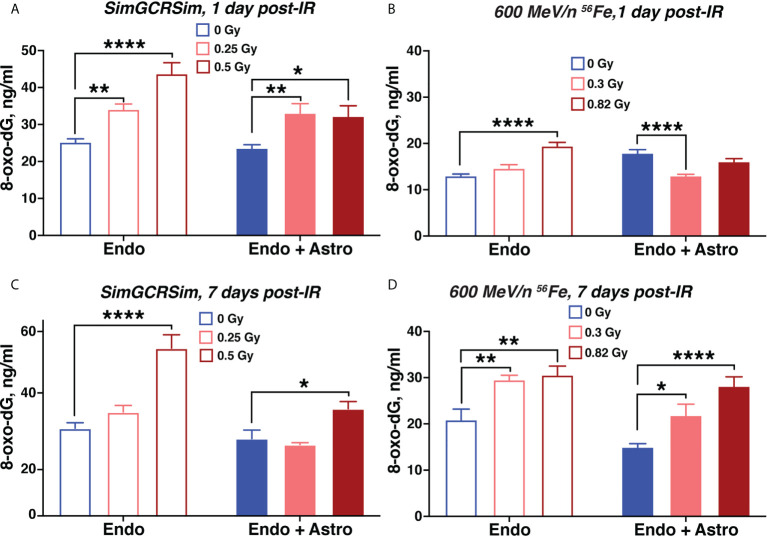
Simulated space radiation induces oxidative stress that is partially mitigated by astrocytes. **(A–D)** Quantification of secreted 8-oxo-dG in supernatants of chips seeded with endothelial cells only (open bars) and endothelial cells + astrocytes (shaded bars) 1 day **(A, B)** and 7 days **(C, D)** after irradiation with SimGCRSim **(A, C)** or 600 MeV/n ^56^Fe **(B, D)** particles. Blue, 0 Gy sham irradiation. Light red, lower dose (0.3 Gy 600 MeV/n ^56^Fe, 0.25 Gy SimGCRSim). Dark red, higher dose (0.82 Gy 600 MeV/n ^56^Fe, 0.5 Gy SimGCRSim). N = 12 chips per condition. Error bars, mean ± SEM. *p < 0.05, **p < 0.01, ****p < 0.0001, 2-way ANOVA, Dunnett’s multiple comparison’s test. Non statistically significant changes are not marked.

We observed a radiation-mediated increase in 8-oxo-dG in chips with endothelial cells alone throughout the time course of 1-7 days after 600 MeV/n ^56^Fe or SimGCRSim irradiation (**Figures 6A–D**). In chips with astrocytes the radiation-mediated oxidative stress was significantly reduced throughout the time course compared with chips with endothelial cells alone indicating a potential antioxidant and radioprotective function of astrocytes during subacute radiation responses. A similar increase in oxidative stress was observed in human endothelial cells, but not astrocytes 3 days after 600 MeV/n ^56^Fe irradiation when the cells were cultured in standard *in vitro* 6-well plates instead of OrganoPlate (**Supplementary Figure 3**).

**Figure 7:**
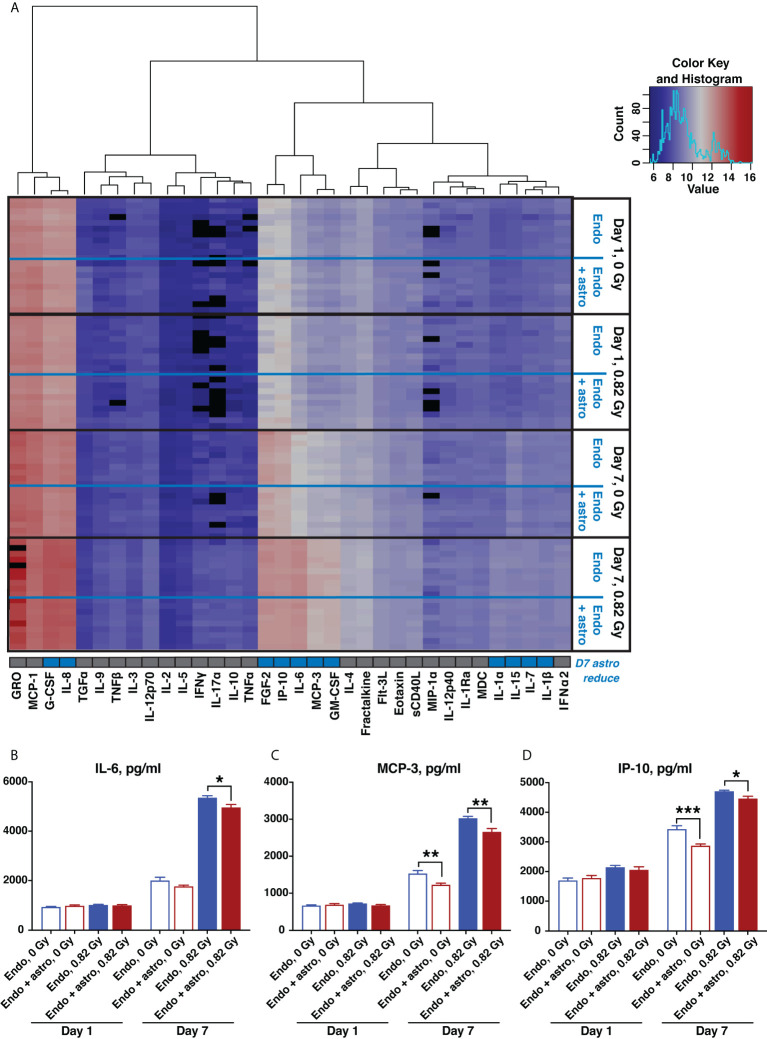
600 MeV/n ^56^Fe radiation stimulates inflammatory cytokine and chemokine production, which is partially mitigated by astrocytes in the subacute phase after irradiation. **(A)** Heatmap of supernatant cytokine and chemokine quantification, pg/ml, log_2_ scale, blue (low) to red (high). Black, no data available. Bottom row, cytokines that are significantly reduced by astrocyte presence 7 days after irradiation (p < 0.05, 2-way ANOVA) marked in blue. **(B–D)** Representative quantification of key inflammatory cytokines and chemokines: IL-6 **(B)**, MCP-3 **(C)** and IP-10 **(D)**, 1 and 7 days after irradiation with 600 MeV/n ^56^Fe particles. Open bars, cytokines and chemokines secreted by endothelial cells only. Shaded bars, cytokines and chemokines secrered by endothelial cells together with astrocytes. Blue, 0 Gy sham irradiation. Dark red, higher dose (0.82 Gy). N = 9-10 chips per condition. Error bars, mean ± SEM. *p < 0.05, **p < 0.01, ***p < 0.001, Student’s t-test. Non statistically significant changes are not marked.

Quantification of human pro- and anti-inflammatory cytokines and chemokines in supernatants from OrganoPlate chips 1 and 7 days after 0.82 Gy 600 MeV/n ^56^Fe irradiation or 0 Gy sham control indicated a major radiation-mediated increase in both pro- and anti-inflammatory cytokines and chemokines by 7 days in all conditions (**Figure 7A**). Comparison between cytokines and chemokines released by astrocytes and endothelial cells together and by endothelial cells alone is marked by set of rows on the right of the heatmap. At that timepoint astrocytes modestly, but significantly reduced the upregulation of multiple pro-inflammatory cytokines and chemokines (**Figure 7A**, row below the heatmap, marked in blue).

Selected pro-inflammatory cytokines and chemokines that were significantly reduced by astrocytes 7 days after irradiation are shown in **Figures 7B–D**. Specifically, IL-6 (**Figure 7B**) is a master regulator of multiple inflammatory cascades after CNS injury (38), while MCP-3 (**Figure 7C**) and IP-10 (**Figure 7D**) respectively act as chemoattractants of monocytes and T lymphocytes (39, 40). Thus, consistent with antioxidant characteristics shown in **Figure 6**, during the subacute stage after irradiation astrocytes adopt a protective phenotype, reducing the oxidative stress and inflammation caused by 600 MeV/n ^56^Fe particles.

To examine astrocytic responses to particle radiation in greater detail, astrocytes were cultured without endothelial cells either in OrganoPlates or in regular cell culture plates and irradiated with equivalent doses of 600 MeV/n ^56^Fe particles. Three days after irradiation, astrocytes in OrganoPlates showed major radiation dose-dependent reduction in selected cytokines and chemokines, including both anti-inflammatory IL-1ra, pro-inflammatory eotaxin, MCP-1 and IP-10, and immune cell proliferation factor FGF2 (**Supplementary Figures 4A-E**). These results indicate a complex astrocytic response to radiation that primarily reduces inflammation, consistently with the role of astrocytes in combined astrocyte-endothelial cell systems (**Figure 7**). Furthermore, astrocytes are highly radiosensitive when cultured without endothelial cells, showing a reduction in cellular health in presence of either low or high doses of 600 MeV/n ^56^Fe particles. Thus, both endothelial cells and astrocytes might be considered to be radioprotective for each other, though the associated mechanisms remain to be investigated in more detail in upcoming studies.

## Discussion

### Novel human model to study the biological effects of space radiation on the blood-brain barrier indicates a complex relationship between astrocytes and endothelial cells

We have developed a novel, high-throughput (96 chips per standard 384-well plate format), human organ-on-a-chip model for investigating BBB impairments caused by deep space radiation. Since the model can be seeded with human primary cells, it is suitable for personalized medicine approaches and to investigate the variability and genomic and epigenetic associations of human radiosensitivity. Meanwhile, the high-throughput nature of our model allows simultaneous detection of multiple outcomes and could be adapted to countermeasure screening and validation.

Here we present the first study utilizing this model to investigate the BBB damage caused by simulated deep space radiation, which is a major health risk for astronauts on upcoming long-duration lunar and Mars missions. To date, research on human CNS effects of space radiation is highly limited and almost exclusively restricted to computational modeling (41–43) and individual cell lines (44). In contrast, our approach, provides a multicellular 3D representation of brain vasculature: vascular endothelial tubes that can be analyzed to quantify the effects of radiation on vascular permeability, and to evaluate the potential regulatory effects of astrocytes by co-culturing them in a neighboring lane of the same chip.

On the other hand, this BBB model is highly simplified, containing only astrocytes and endothelial cells out of all *in vivo* BBB components. In future experiments it will be expanded to a 3-lane system to include other cell types such as neurons (19), perivascular pericytes, microglia and peripheral monocytes in the vascular channel (45), Increased complexity of the model will allow us to understand the role of astrocytes in regulating cell extravasation and investigate the potential structural and regulatory functions of pericytes in radiation responses, which currently remain unknown.

We observed that simulated deep space radiation induces vascular permeability, damages tight junctions, causes oxidative stress and inflammatory cytokine production. In addition, during the acute phase (day 1) after irradiation, astrocytes exacerbate vascular leakiness, while throughout both acute and subacute phases (days 1 – 7) after irradiation, astrocytes become reactive and adopt an antioxidant, anti-inflammatory regulatory phenotype. Our results emphasize the complex relationship between cerebral vascular endothelial cells and astrocytes: endothelial cells respond to simulated deep space radiation by changes in permeability, tight junctions, oxidative stress and cytokine and chemokine secretion, and these processes are modulated by astrocytes. Since astrocyte activation is delayed to 3 – 7 days post irradiation and follows primarily endothelial responses, to acute irradiation. it is possible that endothelial cells regulate astrocytic responses as well, creating a two-way system. Nonetheless, astrocytes might be a more suitable cell-specific target for countermeasure development, especially during the subacute responses to radiation, due to their observed capacity to limit space radiation-mediated endothelial damage.

The specific mechanisms regulating astrocyte activation by particle irradiation and their radioprotective functions remain unknown. When irradiated without the presence of other cells, astrocytes do not show an increase in oxidative stress compared to endothelial cells, and tend to reduce the expression of major pro-inflammatory chemokines, such as IP-10 and MCP-1. This response directly opposes the effects of space radiation on IP-10 and MCP-3 expression in endothelial cells, and might contribute to astrocytic mitigation of the expression of inflammatory chemokines in a combined endothelial cell-astrocyte BBB model. However, astrocyte responses to radiation also involve cellular damage and a reduction of anti-inflammatory IL-1ra expression, suggesting that the mechanisms underlying astrocyte-mediated BBB protection require more in-depth analysis, such as transcriptomics and proteomics that are planned in future studies.

It is difficult to assess the physiological relevance of the radioprotective effects of astrocytes in our model given the current state of research. There have been limited studies on human CNS model responses to simulated deep space radiation, and most of them do not focus on astrocytes and BBB. Meanwhile, the last humans exposed to deep space radiation *in vivo* were Apollo astronauts, but due to the short flight duration the absorbed radiation doses were orders of magnitude lower than those expected on lunar and Mars missions. While prolonged (6 month – 1 year) missions on the International Space Station might provide more information, there are no molecular/cellular astronaut CNS data available to date, while MRI findings cannot be directly related to changes at the cellular level. Thus, a combination of rodent *in vivo* and increasingly complex human organ *in vitro* models, in true spaceflight and its analogs, might serve as the best currently available approach to improving our understanding of human CNS health risks in deep space exploration.

### Human BBB responses to simulated space radiation overlap with rodent *in vivo* damage from ionizing radiation and combined exposures to spaceflight stressors

Previous studies have reported similar observations to our findings regarding astrocyte activation, high levels of astrocytic regulator of vascular permeability AQP4, and reduction in endothelial tight junction protein ZO1 in brain and retinal samples from spaceflown mice (33, 46), and in response to a combination of simulated microgravity and low dose rate gamma irradiation (47). This overlap suggests that the effects of spaceflight are strongly mediated by ionizing radiation, which might become a serious health risk on deep space missions. In addition, it indicates a potential synergistic effect of combined exposures to microgravity and radiatios.

Rodent astrocytes have previously been shown to respond to ^56^Fe irradiation by increased GFAP expression and oxidative stress (48, 49). These results are consistent with our observations in human astrocytes, although the experimental design involved significantly higher doses, which are less representative of anticipated spaceflight exposures. Similarly, rodent responses to simulated GCRs or their components, especially 600 MeV/n ^56^Fe particles, include neuroinflammation as demonstrated by microglia activation in the brain (5), which is consistent with our observed results of pro-inflammatory cytokine and chemokine secretion.

However, to our knowledge, there are no published studies on multicellular *BBB* responses to simulated GCRs or their components in either human or animal models. Thus, our study provides the first step towards understanding the role of astrocytes in regulating BBB, and possibly, other CNS responses to simulated deep space radiation. Furthermore, considering that both spaceflight and simulated deep space radiation dysregulate the immune system (50), the observed disruption of the endothelial cell barrier might exacerbate the neurological damage by encouraging the influx of peripheral immune cells into brain parenchyma, which deserves further investigation.

### Human BBB model responses to simulated deep space radiation resemble neurological disorders

The time course of OrganoPlate BBB responses to radiation including oxidative stress, inflammation and endothelial barrier damage, as well as astrocyte activation combined with their antioxidant and anti-inflammatory properties, strongly resembles CNS responses to traumatic brain injury and stroke (29). These terrestrial conditions are similarly characterized by a complex astrocyte response, including both pro- and anti-inflammatory functions, switching from acute pro-inflammatory to subacute anti-inflammatory phase that is accompanied by GFAP^+^ astrocyte scar formation. The subacute outcomes of astrocyte activation, oxidative stress, vascular endothelial barrier damage and inflammation also overlap with CNS aging and neurodegeneration (51, 52).

Although it is not fundamentally surprising that BBB responses to space and terrestrial stressors are similar, identifying the specific characteristics of space radiation-mediated impairments and comparing them to terrestrial disorders allows potential repurposing of FDA-approved therapeutics as spaceflight countermeasures against CNS damage. We have previously applied a similar approach to agnostically identify a human disease signature that resembles spaceflight-mediated changes in gene expression *in vivo* (53). On the other hand, the similarities suggest that particle radiation could also be a model of terrestrial CNS diseases, especially aging, which might in turn accelerate aging research.

### Further human CNS organ-on-a-chip model development for space biology research

In addition to gradually expanding our simplified human model to include other CNS components, such as neurons, pericytes, microglia and circulating immune cells, it will be important to compare it to the *in vitro* effects in mouse models, which will require developing an equivalent organ-on-a-chip model seeded with primary mouse instead of human cells. Our model currently has the advantage of analyzing the acute and subacute effects of radiation, while most *in vivo* studies begin at least 1 month after irradiation due to time constraints of behavioral experiments (3). On the other hand, the volumetric limitations of our model allow for up to 1 month of experiments (19). Therefore, for the most comprehensive study it will be important to complement them with longer-lasting mouse and human CNS organoids (54).

Furthermore, all experimental paradigms of irradiation with GCRs or their charged particle components are limited to acute, high dose rate irradiation, when the entire dose is either delivered at once or in fractions over multiple days. This method does not accurately replicate the space environment, where astronauts will be exposed to continuous low dose rate radiation. However, biological experiments with chronic, low dose rate charged particle radiation are currently not possible, indicating the need for a dedicated radiation setup to imitate the space environment. On the other hand, chronic low dose rate exposures to neutrons and gamma rays have been developed (55, 56), and CNS effects of chronic gamma irradiation similarly include oxidative stress and inflammatory responses *in vivo* (47, 56, 57). In addition, chronic neutron irradiation causes neuronal damage (58), but its neurovascular and neuroimmune outcomes remain to be investigated.

Finally, in comparison to *in vivo* vertebrate models, organ-on-a-chip models are comparatively low footprint, high throughput and could theoretically be operated in an automated manner. These characteristics make them particularly suitable for missions beyond low-Earth orbit, such as lunar orbit and surface, which will have limited crew time for biological experiments. Thus, developing new engineering approaches to automate both the maintenance of this or similar models and the sampling and analysis of outputs is highly relevant for space biology research, and could also be utilized to facilitate the validation of terrestrial therapeutics.

*In summary*, we have developed a 3D simplified high-throughput, human BBB model and utilized it to investigate the effects of deep space radiation. We observed that simulated GCRs and their ^56^Fe particle components increased vascular permeability, damaged tight junctions, activated astrocytes and caused oxidative stress and the production of inflammatory cytokines during the first week after irradiation. Astrocytes played a dual regulatory role, primarily adopting a radioprotective scar-like phenotype by 7 days after irradiation. Thus, space radiation-mediated astrocyte and endothelial damage resembles CNS injuries and merits further studies to identify the overlapping mechanisms and countermeasures and adapting this model for flight payloads.

## Data availability statement

The raw data supporting the conclusions of this article will be made available by the authors, without undue reservation.

## Author contributions

SV designed and performed experiments, analyzed data, prepared figures and edited the manuscript. EP performed experiments, analyzed data and edited the manuscript. SM designed and performed experiments and analyzed data and edited the manuscript. CJ performed experiments and edited the manuscript. VB performed experiments. SVC edited manuscript, provided intellectual advice and provided funding for the study. EC conceived experiments, performed experiments, analyzed data, prepared figures, wrote/prepared the manuscript and provided funding for the study. All authors contributed to the article and approved the submitted version.

## Funding

The research has been funded by National Aeronautics and Space Administration (NASA) Ames Research Innovation Awards to EC and SVC and by National Aeronautics and Space Administration (NASA) Human Research Program Omnibus Award # 80JSC018N0001 to EC.

## Acknowledgments

We thank Dr. Kristin Bircsak, Dr. Nienke Wevers and the Mimetas. Inc. team for providing training on OrganoPlates. We also thank the National Aeronautics and Space Administration (NASA) Space Radiation Laboratory team at the Brookhaven National Laboratory, especially Dr. Peter Guida and Dr. Adam Rusek, for supporting the simulated deep space radiation experiments.

## Conflict of interest

Author VB is employed by Bionetics Corporation.

The remaining authors declare that the research was conducted in the absence of any commercial or financial relationships that could be construed as a potential conflict of interest.

## Publisher’s note

All claims expressed in this article are solely those of the authors and do not necessarily represent those of their affiliated organizations, or those of the publisher, the editors and the reviewers. Any product that may be evaluated in this article, or claim that may be made by its manufacturer, is not guaranteed or endorsed by the publisher.

## Supplementary material

Supplementary Figure 1 Ionizing radiation causes vascular permeability in OrganoPlate model. **(A–D)** Mean relative fluorescence **(A, C)** and quantification of area under the curve **(B, D)** of chips seeded with endothelial cells 4 hours – 1 week after 1 Gy X-ray irradiation. **(A). (B)**, TRITC-conjugated 155 kDa dextran. N = 6 chips/condition. **(C, D)**, FITC-conjugated 40 kDa dextran. Closed bars, hCMEC/D3 endothelial cells. Open bars, PBMEC endothelial cells. Blue, 0 Gy sham irradiation. Red, 1 Gy X-ray. Error bars, mean ± SEM. 2-way ANOVA: ****p < 0.0001, radiation for hCMEC/D3, 155 kDa. **p < 0.01, radiation for PBMEC, 155 kDa; **p < 0.01, radiation for hCMEC/D3, 40 kDa. ****p < 0.0001, radiation for PBMEC, 40 kDa. **(E, F).** Quantification of area under the curve of chips seeded with endothelial cells and astrocytes 24 and 72 hours after 600 MeV/n ^56^Fe irradiation. TRITC-conjugated 155 kDa dextran. **(E),** hCMEC/D3 endothelial cells. **(F),** PBMEC endothelial cells. Open bars, endothelial cells only. Shaded bars, endothelial cells + astrocytes. Blue, 0 Gy sham irradiation. Light red, lower dose (0.3 Gy). Dark red, higher dose (0.82 Gy). N = 4-6 chips/condition. Error bars, mean ± SEM. 2-way ANOVA: *p < 0.05, interaction, **p < 0.01, astrocyte presence for hCMEC/D3, 24 h, *p < 0.05, radiation, ****p < 0.0001, astrocyte presence for hCMEC/D3, 72 h; ****p < 0.0001, astrocyte presence for PBMEC, 24 h, ****p < 0.0001, astrocyte presence for PBMEC, 72 h. Non statistically significant changes are not marked.

Supplementary Figure 2 Vascular endothelial responses to SimGCRSim irradiation. **(A–D).** Quantification of endothelial ZO1 **(A, B)** and PECAM1 **(C, D)** immunofluorescence 1 day after irradiation. **(E–H).** Quantification of endothelial ZO1 **(E, F)** and PECAM1 **(G, H)** immunofluorescence 7 days after irradiation. Open bars, chips with endothelial cells only. Shaded bars, chips with endothelial cells and astrocytes. Blue, 0 Gy sham irradiation. Light red, lower dose (0.25 Gy). Dark red, higher dose (0.5 Gy). N = 12 areas from 6 chips per condition. Error bars, mean ± SEM. *p < 0.05, **p < 0.01, ***p < 0.001, ****p < 0.0001, 1-way ANOVA, Dunnett’s multiple comparisons test. Non statistically significant changes are not marked.

Supplementary Figure 3 Ionizing radiation causes oxidative stress in endothelial cells and astrocytes. Secreted 8-oxo-dG 3 days post irradiation with 600 MeV/n ^56^Fe particles, cells cultured in 6-well plates instead of OrganoPlates. Open bars, endothelial cells. Shaded bars, astrocytes. N = 6 chips/condition. Blue, 0 Gy sham irradiation. Light red, lower dose (0.3 Gy). Dark red, higher dose (0.82 Gy). Error bars, mean ± SEM. **p < 0.01, 1-way ANOVA. Non statistically significant changes are not marked.

Supplementary Figure 4 Ionizing radiation affects astrocytic cytokine and chemokine secretion and causes cellular damage. **(A–E)**, quantification of key inflammatory cytokines and chemokines: IL-1ra **(A)**, Eotaxin **(B)**, MCP-1 **(C)**, IP-10 **(D)** and FGF2 **(E)** secreted by astrocytes cultured in OrganoPlates, 3 days after irradiation with 600 MeV/n ^56^Fe particles. Blue, 0 Gy sham irradiation. Pink, lower dose (0.3 Gy). Dark red, higher dose (0.82 Gy). N = 5-6 chips per condition. Error bars, mean ± SEM. *p < 0.05, **p < 0.01, Kruskal-Wallis test with Dunn’s correction for multiple comparisons. Non statistically significant changes are not marked. F. Representative brightfield images of astrocytes cultured in regular 6-well plates, 3 days after irradiation with 600 MeV/n ^56^Fe particles. Scale bar, 100μm.
