# Supplementary figures and images for: Astrocytes regulate vascular endothelial responses to simulated deep space radiation in a human organ-on-a-chip model
Source: Front Immunol. 2022 Aug 30;13:864923. doi: 10.3389/fimmu.2022.864923 (PMC9580499; doi:10.3389/fimmu.2022.864923)

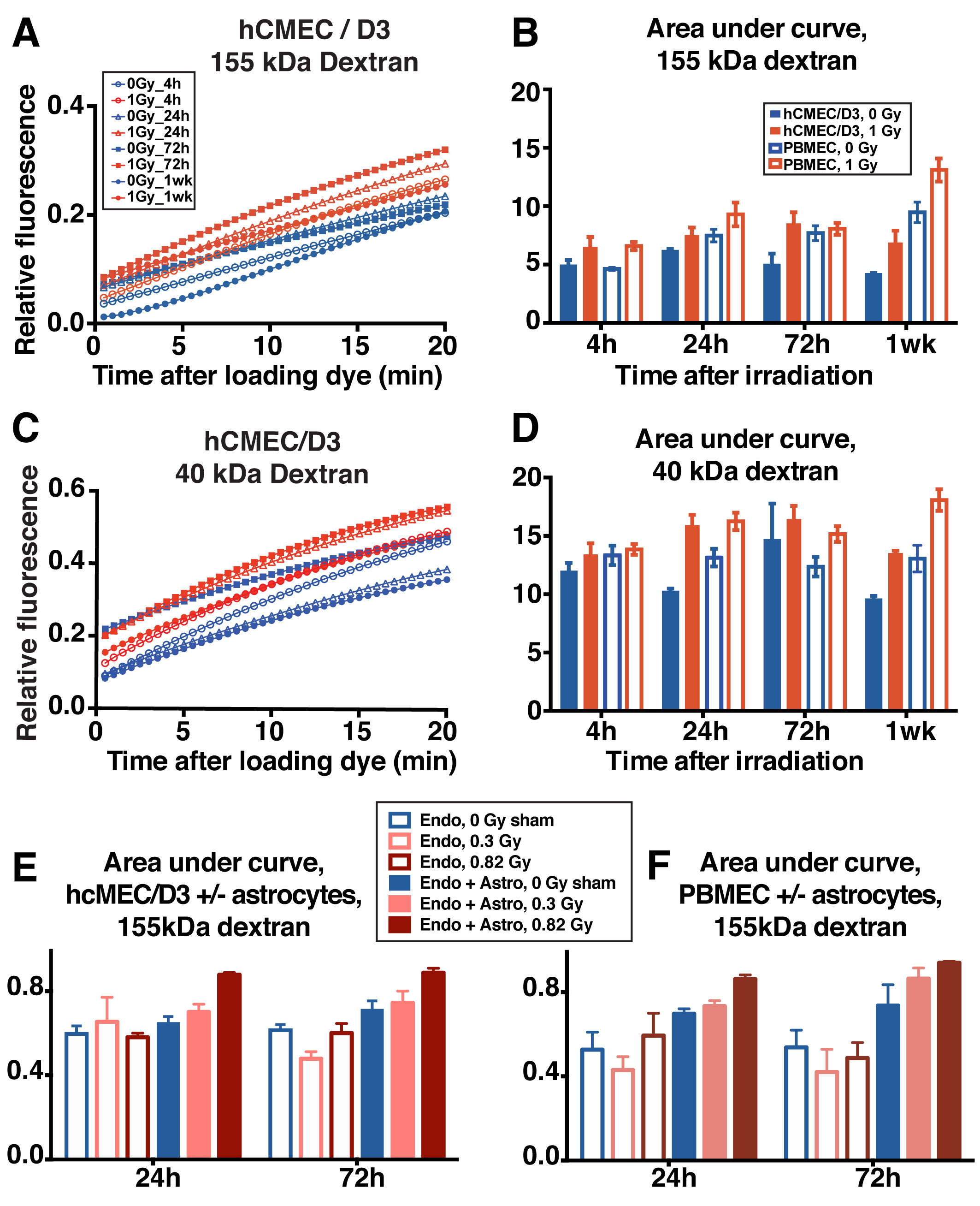

Supplement: Supplementary Figure 1 — Ionizing radiation causes vascular permeability in OrganoPlate model. (A–D) Mean relative fluorescence (A, C) and quantification of area under the curve (B, D) of chips seeded with endothelial cells 4 hours – 1 week after 1 Gy X-ray irradiation. (A). (B), TRITC-conjugated 155 kDa dextran. N = 6 chips/condition. (C, D), FITC-conjugated 40 kDa dextran. Closed bars, hCMEC/D3 endothelial cells. Open bars, PBMEC endothelial cells. Blue, 0 Gy sham irradiation. Red, 1 Gy X-ray. Error bars, mean ± SEM. 2-way ANOVA: ****p < 0.0001, radiation for hCMEC/D3, 155 kDa. **p < 0.01, radiation for PBMEC, 155 kDa; **p < 0.01, radiation for hCMEC/D3, 40 kDa. ****p < 0.0001, radiation for PBMEC, 40 kDa. (E, F). Quantification of area under the curve of chips seeded with endothelial cells and astrocytes 24 and 72 hours after 600 MeV/n 56Fe irradiation. TRITC-conjugated 155 kDa dextran. (E), hCMEC/D3 endothelial cells. (F), PBMEC endothelial cells. Open bars, endothelial cells only. Shaded bars, endothelial cells + astrocytes. Blue, 0 Gy sham irradiation. Light red, lower dose (0.3 Gy). Dark red, higher dose (0.82 Gy). N = 4-6 chips/condition. Error bars, mean ± SEM. 2-way ANOVA: *p < 0.05, interaction, **p < 0.01, astrocyte presence for hCMEC/D3, 24 h, *p < 0.05, radiation, ****p < 0.0001, astrocyte presence for hCMEC/D3, 72 h; ****p < 0.0001, astrocyte presence for PBMEC, 24 h, ****p < 0.0001, astrocyte presence for PBMEC, 72 h. Non statistically significant changes are not marked. [file Image_1.jpeg]

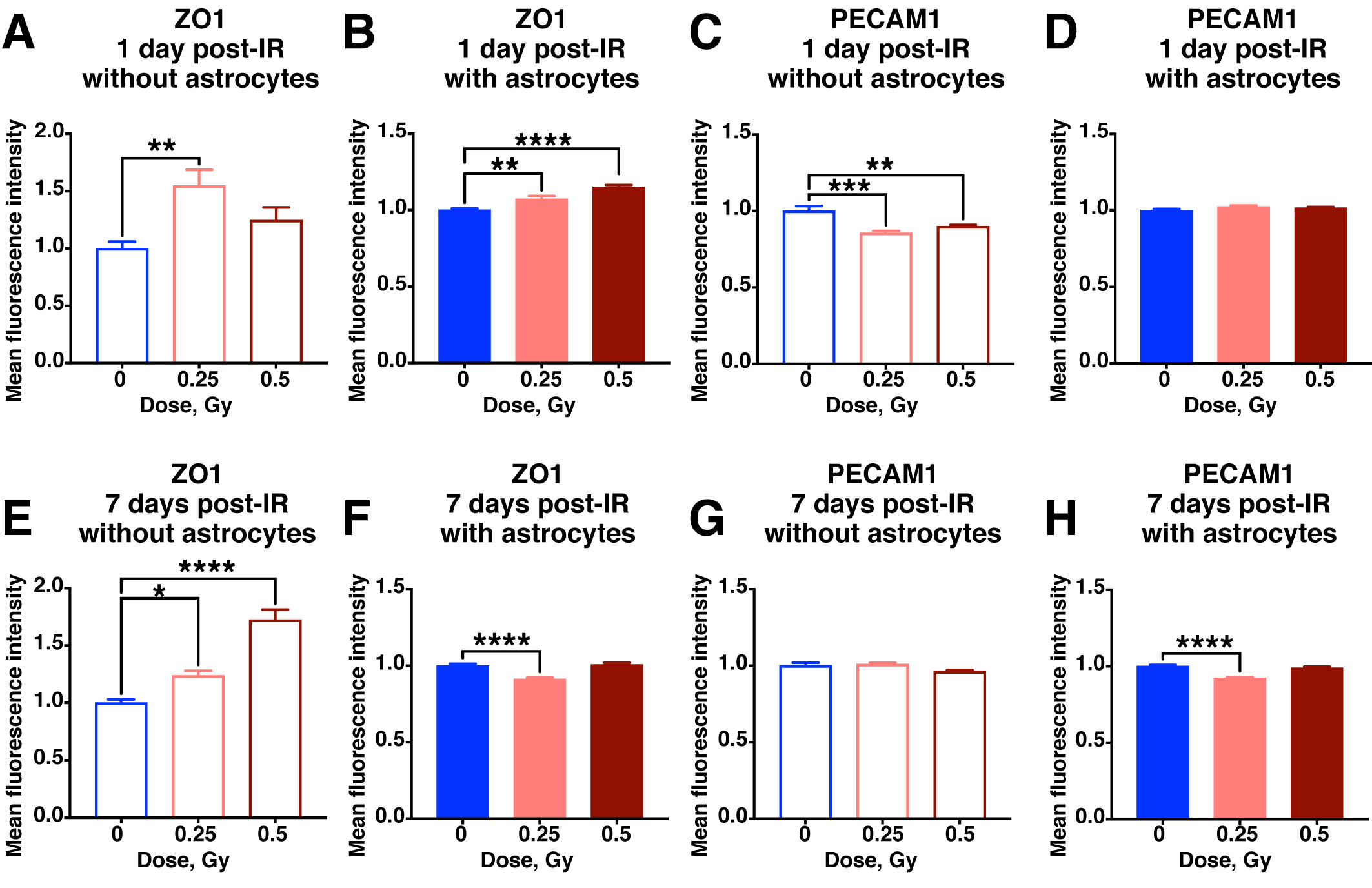

Supplement: Supplementary Figure 2 — Vascular endothelial responses to SimGCRSim irradiation. (A–D). Quantification of endothelial ZO1 (A, B) and PECAM1 (C, D) immunofluorescence 1 day after irradiation. (E–H). Quantification of endothelial ZO1 (E, F) and PECAM1 (G, H) immunofluorescence 7 days after irradiation. Open bars, chips with endothelial cells only. Shaded bars, chips with endothelial cells and astrocytes. Blue, 0 Gy sham irradiation. Light red, lower dose (0.25 Gy). Dark red, higher dose (0.5 Gy). N = 12 areas from 6 chips per condition. Error bars, mean ± SEM. *p < 0.05, **p < 0.01, ***p < 0.001, ****p < 0.0001, 1-way ANOVA, Dunnett’s multiple comparisons test. Non statistically significant changes are not marked. [file Image_2.jpeg]

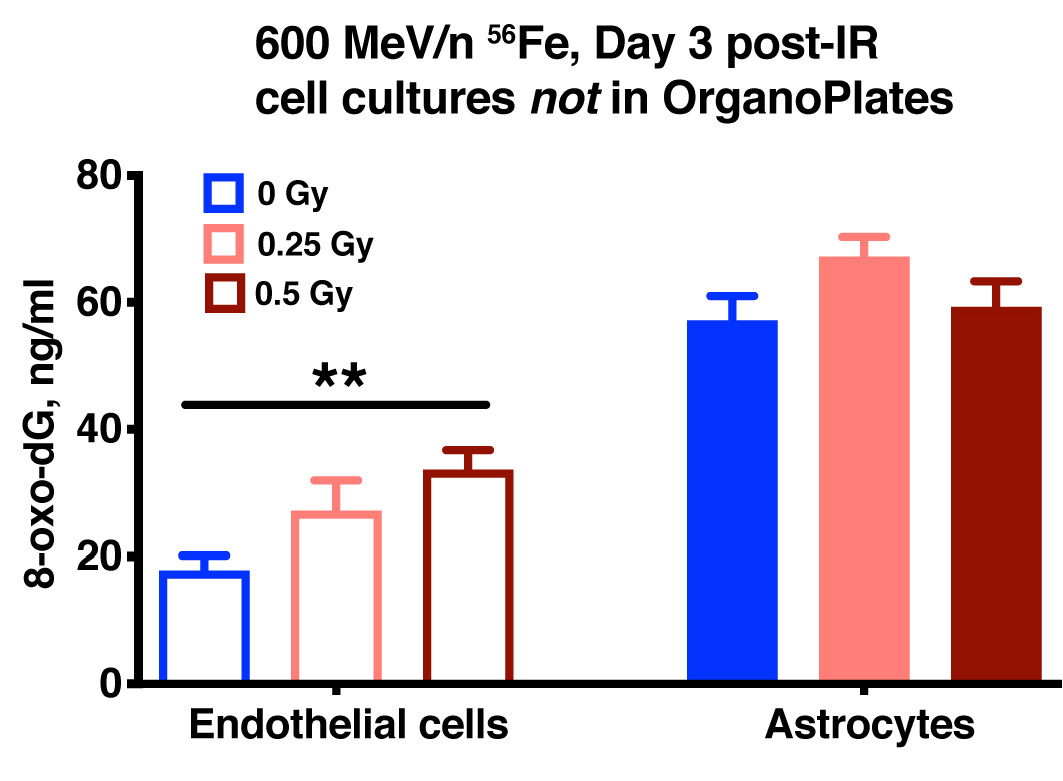

Supplement: Supplementary Figure 3 — Ionizing radiation causes oxidative stress in endothelial cells and astrocytes. Secreted 8-oxo-dG 3 days post irradiation with 600 MeV/n 56Fe particles, cells cultured in 6-well plates instead of OrganoPlates. Open bars, endothelial cells. Shaded bars, astrocytes. N = 6 chips/condition. Blue, 0 Gy sham irradiation. Light red, lower dose (0.3 Gy). Dark red, higher dose (0.82 Gy). Error bars, mean ± SEM. **p < 0.01, 1-way ANOVA. Non statistically significant changes are not marked. [file Image_3.jpeg]

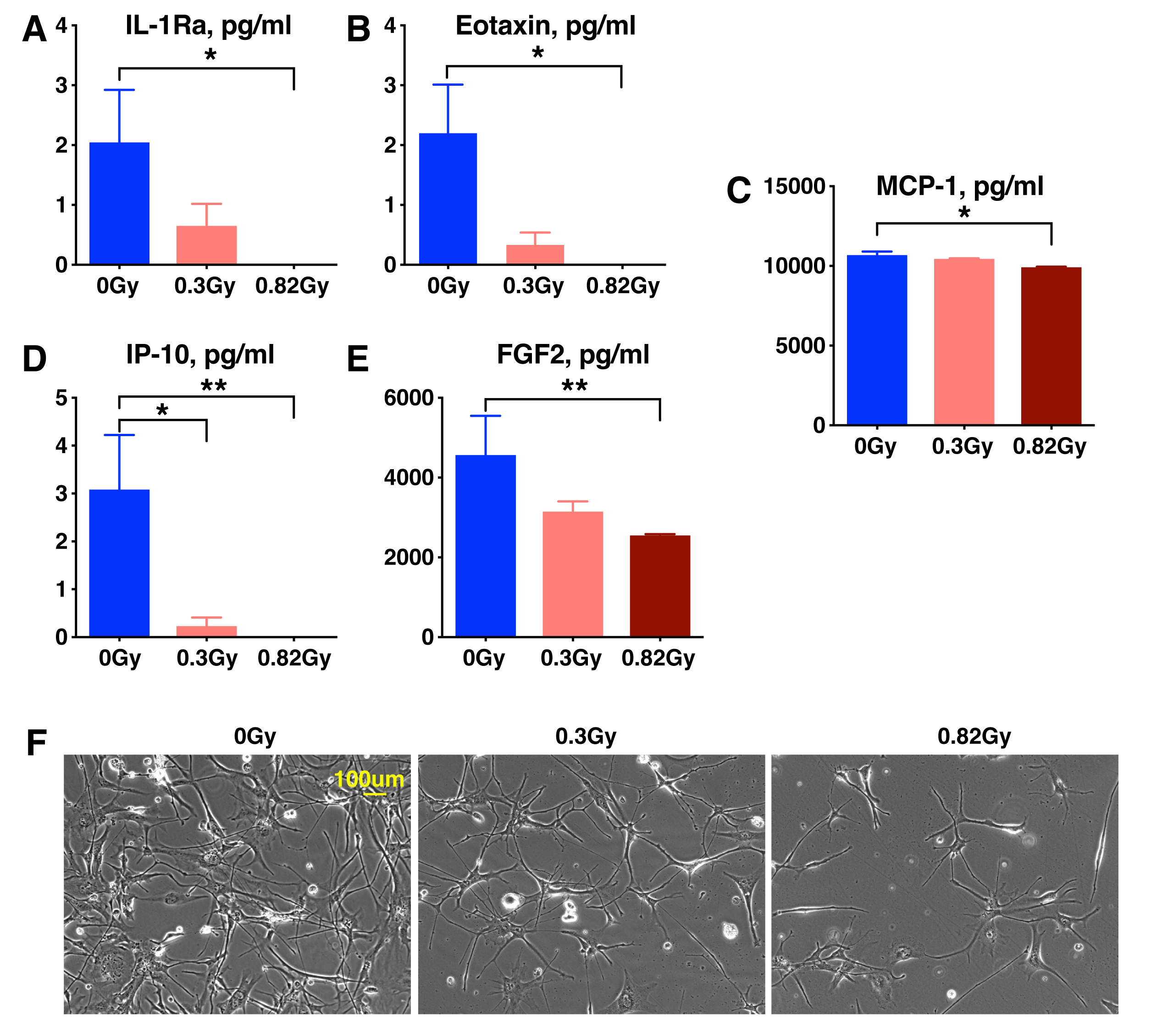

Supplement: Supplementary Figure 4 — Ionizing radiation affects astrocytic cytokine and chemokine secretion and causes cellular damage. (A–E), quantification of key inflammatory cytokines and chemokines: IL-1ra (A), Eotaxin (B), MCP-1 (C), IP-10 (D) and FGF2 (E) secreted by astrocytes cultured in OrganoPlates, 3 days after irradiation with 600 MeV/n 56Fe particles. Blue, 0 Gy sham irradiation. Pink, lower dose (0.3 Gy). Dark red, higher dose (0.82 Gy). N = 5-6 chips per condition. Error bars, mean ± SEM. *p < 0.05, **p < 0.01, Kruskal-Wallis test with Dunn’s correction for multiple comparisons. Non statistically significant changes are not marked. F. Representative brightfield images of astrocytes cultured in regular 6-well plates, 3 days after irradiation with 600 MeV/n 56Fe particles. Scale bar, 100μm. [file Image_4.jpeg]
